# Dose‐effect relation between regular consumption of 100% cocoa powder and blood pressure in young, healthy black Africans

**DOI:** 10.14814/phy2.15070

**Published:** 2021-10-21

**Authors:** Edwige Balayssac‐Siransy, Soualiho Ouattara, Kotchi Joël Michée Boka, Hugues Ahiboh, Téniloh Augustin Yéo, Paule‐Denise Yapo, Aya Liliane Kondo, Walamitien Cyrille Touré, Kotchi Fabrice Edé, Cyrille Serges Dah, Pascal Bogui

**Affiliations:** ^1^ Laboratoire de Physiologie et d’Explorations Fonctionnelles Unité de Formation et de Recherche en Sciences Médicales Université Félix Houphouët‐Boigny Abidjan Côte d’Ivoire; ^2^ Service des Explorations Fonctionnelles Centre hospitalier universitaire de Yopougon Abidjan Côte d’Ivoire; ^3^ Laboratoire de Biochimie Unité de Formation et de Recherche en Sciences Pharmaceutiques et Biologiques Université Félix Houphouët‐Boigny Abidjan Côte d’Ivoire; ^4^ Service des Explorations Fonctionnelles Centre hospitalier universitaire de Cocody Abidjan Côte d’Ivoire

**Keywords:** black African, blood pressure, cocoa, dose‐effect

## Abstract

**Background:**

Some previous works have focused on dose‐response relationship between cocoa consumption and blood pressure in Caucasians. As black subjects have lower nitric oxide bioavailability, the aim of this work was to determine the dose‐effect relation between cocoa and blood pressure in black Africans.

**Method:**

One hundred and thirty healthy black African males aged 18–30 were randomly assigned into four groups: three groups consuming 10 g, 5 g, or 2 g of cocoa powder daily for three weeks and one control group that did not consume cocoa. Systolic blood pressure (SBP), diastolic blood pressure (DBP), and heart rate (HR) were measured on day 1 (D1, before any subject consumed cocoa), D8, D15, and D22. Means of the parameters at each of the four visits and changes of the means were compared among the groups.

**Results:**

Significant decrease in SBP was noted in consumers of 10 g compared to controls in the 1st week, and compared to consumers of 2 g in the 2nd and 3rd weeks of follow‐up. Means and changes of DBP were statistically similar among the four groups.

**Conclusion:**

Among our cohort, decrease in SBP was significantly greater in the heavy cocoa consumer group (10 g) compared to the low consumer group (2 g), but there was no statistically significant difference when compared with the intermediate consumer group (5 g). The dose‐response relationship between cocoa consumption and changes in SBP was not linear. No relationship was found between cocoa consumption and DBP.

## Graphical abstract

Some previous works have focused on the dose‐response relationship between cocoa consumption and blood pressure and found that the highest doses of cocoa induced the greatest drops in blood pressure. Studies have been carried out in Caucasians in whom the bioavailability of nitric oxide, which explains the effect of cocoa on blood pressure, is better than in black subjects. Carried out over three weeks among healthy black Africans, our study found significant reduction of SBP in consumers of largest amount of cocoa with no linear relationship between cocoa doses and SBP variations.

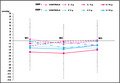

## 1 INTRODUCTION

High blood pressure is an important public health burden in all populations (Kearney et al., 2005; Risk Factor Collaboration and (NCD‐RisC), 2017). The risk of complications increase with arterial blood pressure (Brown et al., 2007). Arterial blood pressure is a physiological parameter determined by cardiac output and peripheral vascular resistance. By acting on these determinants, various factors can reduce blood pressure and its complications. Flavanols, which are flavonoid‐type organic molecules of the polyphenol family (Arora et al., 2019; Ross & Kasum, 2002), are thought to lead to decrease in vascular resistance by increasing the endothelial production of nitric oxide (NO). NO, by its vasodilatory action, induces a drop in blood pressure (Hollenberg et al., 2004). Flavanols are found in varying amounts in fruits and vegetables (Amiot et al., 2012). Cocoa, fruit of the cocoa tree, has been recognized for several years for its richness in flavanols (Ding et al., 2006; Steinberg et al., 2003). Various authors (Balayssac‐Siransy et al., 2018; Hauhouot‐Attoungbré et al., 2011; Ried et al., 2010, 2017) have thus been interested in the effects of regular cocoa consumption on systolic and diastolic blood pressures. The results of their work, which essentially compared only two groups (consumers of small doses of cocoa or controls without cocoa versus consumers of high flavanols), showed a greater reduction in BP with high flavanols whereas some other groups reported a smaller reduction after high flavanols ingestion. In studies carried out in normotensive patients, this difference was between −7.1 and 6.29 mmHg for systolic blood pressure (SBP) and between −7.6 and 1.5 mmHg for diastolic blood pressure (DBP) (Balayssac‐Siransy et al., 2018; Ried et al., 2017; Siransy‐Balayssac et al., 2021). Therefore, some authors found decrease in blood pressure, while others found increase in blood pressure in consumers of high doses of cocoa, compared to control subjects.

These differences in results may be due to differences in methodologies, the type of product consumed, the duration of consumption, and the characteristics of the study population (Ried et al., 2017; Taubert et al., 2007). Indeed, the various researches were carried out using different cocoa‐based products, in the form of chocolate or drink, with or without sugar, and it is important to note that sugar inhibits the vasodilatory effects of flavanols (Ried et al., 2017). Subjects consumed the products over different periods, ranging from 2 to 18 weeks. The populations of these studies were often mixed gender (Ried et al., 2010, 2017), and changes in blood pressure with the menstrual cycle have been described (Balayssac‐Siransy et al., 2014; Moran et al., 2000). In addition, the previous studies focused on populations of different ages, and a greater drop in blood pressure has been described in subjects under the age of 50 years (Ried et al., 2017). Subjects included in the studies had different levels of basal blood pressure. According to Ried et al. (Ried et al., 2010, 2017), drops in blood pressure were greater in patients who had higher blood pressures at entry into the study. The populations were mainly Caucasian (Ried et al., 2010, 2017) and rarely black Africans (Balayssac‐Siransy et al., 2018; Hauhouot‐Attoungbré et al., 2011). However, various studies have found lower level of NO and its precursor L‐Arginine in black subjects compared to Caucasians (Glyn et al., 2012; Kalinowski et al., 2004; Ozkor et al., 2014). It is therefore difficult, on the basis of different methodologies of these previous works, to determine the dose‐response relationship between cocoa consumption and changes in blood pressure. Only few studies (Davison et al., 2010; Mastroiacovo et al., 2015) have investigated this dose‐effect relationship by constituting three or four groups of consumers based on the daily doses of flavanols consumed. The studies were performed in Caucasian, mixed gender, age‐varying populations with different levels of blood pressure upon entry into the study. A significantly greater decrease in blood pressure was found with larger doses of cocoa, without evidence of a dose‐response relationship (Davison et al., 2010). The goal of our work was to determine over three weeks the dose‐effect relationship between regular consumption of different doses (2, 5, and 10 g) of the same 100% cocoa powder and blood pressures among young, healthy black African male subjects.

## 2 MATERIALS AND METHODS

### 2.1 Ethics approval

This study was approved by the Ethics Committee of the University Teaching Hospital of Yopougon (Abidjan, Côte d`Ivoire) and followed the guidelines of the Declaration of Helsinki. All patients were informed about the purpose of the study and gave their written consent.

### 2.2 Study population

#### 2.2.1 Target population

The population consisted of volunteer students from Félix Houphouët‐Boigny University (Abidjan, Côte d`Ivoire).

#### 2.2.2 Selection criteria

For this study, students were black Africans, males, aged between 18 and 30 years, with body mass index (BMI) between 18.5 and 29.9 kg/m². Smoking; regular alcohol consumption; dyslipidemia; diabetes; arterial hypertension; cardiovascular, respiratory, hematological, or infectious symptomatology or disease; unexplained fatigue; physical activity score greater than 35 points (corresponding to very active subjects) according to Ricci and Gagnon scoring system; recent or current intake of a drug that can modify SBP or DBP; and regular consumption of any cocoa‐based product or product rich in flavonoids (fruits, nuts, coffee, tea, or wine) were non‐inclusion criteria.

#### 2.2.3 Study forms

Presence of inclusion and non‐inclusion criteria was assessed with a survey sheet which was organized into two parts: a questionnaire and a record of anthropometric measurements. The questionnaire portion was filled by the participant in the presence of an investigator. Height and weight were measured with the same method used in a previous study (Siransy‐Balayssac et al., 2021).

#### 2.2.4 Sample

**FIGURE 1:**
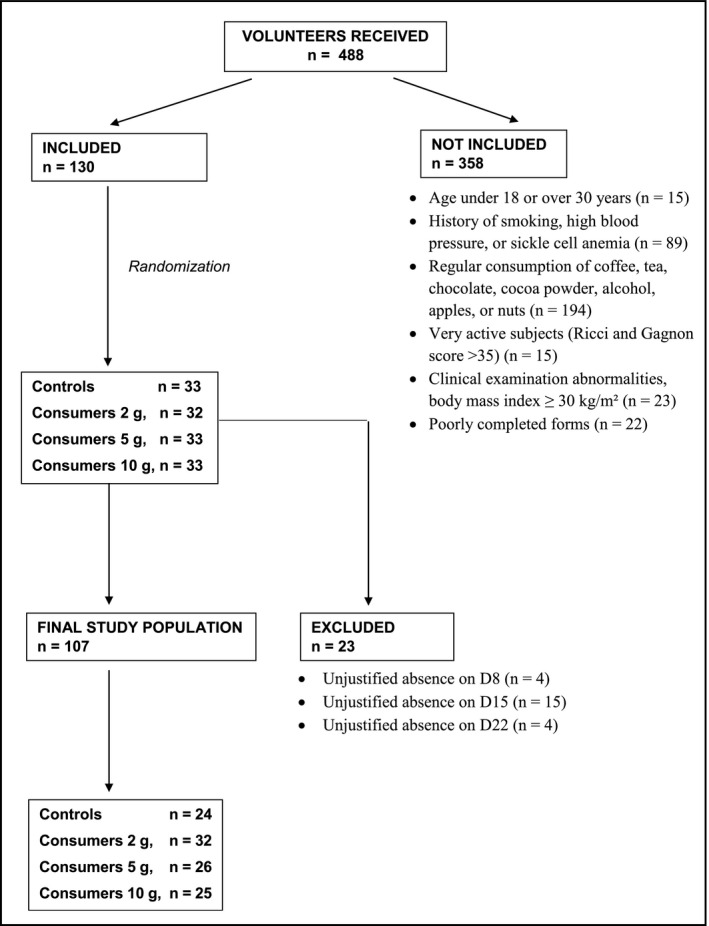
Reasons for non‐inclusion and exclusion of subjects from the study

Among 488 volunteer students, 130 were selected according to our selection criteria (Figure 1), and these were then randomized into four groups: 32 in the control group, 32 in the 2 g cocoa consumer group, 33 in the 5 g cocoa consumer group, and 33 in the 10 g cocoa consumer group. During the three weeks of the study, participants were withdrawn if their SBP dropped lower than 90 mmHg, they took a drug that can modify blood pressure, they were unable to attend the appointment, they withdrew from the study, they required medical intervention, they experienced major stress, they suffered from adverse effects linked to the consumption of cocoa powder (allergy, digestive disorders, disgust, or nervousness), or if they were absent without justification. Based on these exclusion criteria, 23 subjects were withdrawn (Figure 1). Therefore, 107 subjects fully completed the study protocol: 24 controls, 32 in the 2 g cocoa consumer group, 26 in the 5 g cocoa consumer group, and 25 in the 10 g cocoa consumer group, with mean ages of 21.4 ± 2.3 years, 21.7 ± 1.8 years, 21.5 ± 1.9 years, and 22 ± 2.6 years (*p* = 0.93) respectively, and mean BMI of 21.5 ± 2 kg/m², 21.3 ± 2 kg/m², 21.9 ± 1.9 kg/m², and 21.6 ± 2.1 kg/m², respectively (*p* = 0.49).

### 2.3 Study material

#### 2.3.1 Equipment

An electronic scale (Exacta type Premium, Germany) was used to measure weight in kilograms. Electronic sphygmomanometers (Omron type M6, Japan) were used to measure SBP, DBP, and heart rate (HR).

#### 2.3.2 Cocoa powder

Three brands of 100% cocoa powder that were available in supermarkets in Abidjan (Côte d’Ivoire) were selected: Tafissa, Nestle, and Ryan's. The analysis of polyphenols and flavonoids in these powders were carried out by the Laboratory of Industrial Processes, Synthesis, Environment and New Energies (LAPISEN) of the National Polytechnic Institute Houphouët‐Boigny of Yamoussoukro (Côte d’Ivoire). The method of Wood et al. (Wood et al., 2002) was used for measuring total polyphenol content. Total flavonoid content was measured according to Marinova et al. (Marinova et al., 2005). Analyses indicated higher flavonoid content in the Tafissa and Nestle powders (Table 1). After considering the availability of a 5‐g stick packaging, the ease of supply in the local market, and the cost of the powder, Tafissa, which is a cocoa powder from the *Forastero* variety, was retained for this study. The sticks used came from the following three batches: PS100‐17 074/17–088 from March 15, 2017 to March 15, 2019; PS100‐17 075/17–131 from March 16, 2017 to March 16, 2019; and PS100‐17 138/17–143 from May 18, 2017 to May 18, 2019.

**TABLE 1:** Polyphenol and flavonoid content in cocoa powders

Cocoa powder brands	Mean ±Standard deviation	
	Polyphenol (mg/g GAE)	Flavonoid (mg/g QE)
Tafissa	295 ± 8	168 ± 3
Nestle	416 ± 11	165 ± 7
Ryans	340 ± 12	111 ± 3

Abbreviations: GAE, gallic acid equivalent, QE, quercetin equivalent.

#### 2.3.3 Sheets

A follow‐up form filled out at each meeting made it possible for participants to notify us about the presence of acute stress, symptoms, or use of medications during the seven days preceding the current visit. A food sheet given to each subject at the beginning of each week was used for daily recording of the types of food consumed and for recording any medication taken. On this sheet, the instructions to be followed were provided, including foods to avoid, and participants were directed to consume cocoa in the morning on an empty stomach, thirty minutes before breakfast.

### 2.4 Study protocol

This was an experimental, prospective, randomized, single‐blind study (the investigators at the measuring station were blinded to the grouping of the participant). The protocol was performed in two steps.

The subjects included in the study were informed about the foods to avoid for the seven days before the date set for their entry into the 2nd step. These included food products that contain significant amount of flavanols (foods made from cocoa, fruits, tea, coffee, nuts, wine, and other alcohols).

The 2nd stage lasted three weeks, during which participants were assessed four times: on day 1 (D1) (before taking cocoa), on D8 (8th day corresponding to the end of the 1st week (W1)), on D15 (15th day corresponding to the end of the 2nd week (W2)), and D22 (22nd day corresponding to the end of the 3rd week (W3)). The follow‐up protocol during this second stage was the same on each of the four visits. Participants were directed to fast for ten hours before arriving for assessment between 7 am and 8 am. Each individual follow‐up sheet was assessed for specific dates of major stress, symptoms, or drug intake. Body weight was measured as described previously and participants were then placed in groups of three, in a seated position (the back and arms supported and the feet on the ground). Each participant faced one of the corners of the quiet, semi‐lit, air‐conditioned room at 22°C. A blood pressure cuff was placed on the non‐dominant arm of each subject. After 5 minutes of rest, the arterial blood pressure was measured according to the recommendations of the European Society of Hypertension and European Society of Cardiology (Mancia et al., 2013). SBP, DBP, and HR were recorded at the 5th, 7th, and 10th minutes of rest. Talking was not allowed during this 10 min. On each of the four visits, each participant occupied the same place (previously numbered) in the same room, and the measurements were made with the same blood pressure monitor (also previously numbered).

After leaving the blood pressure measurement room, the participants were received, in turn, in another room by a single investigator appointed for the duration of the study. Each subject was always alone with the investigator. During the first visit (D1), this investigator communicated the study group (consumer or control) to each participant, while specifying that this information should not be communicated to a third person (neither participant nor investigator). According to the subject group, the investigator then diluted 2 g (preconditioned in tubes and containing 336 mg of flavonoids), 5 g (corresponding to 1 stick containing 840 mg of flavonoids), or 10 g (corresponding to 2 sticks containing 1,680 mg of flavonoids) of cocoa powder in water, and the participant consumed it in the investigator's presence. The investigator then gave the participant the other doses (of 2, 5, or 10 g) of cocoa powder for the remaining six days of the week, together with a food sheet to be completed by the participant. The control participants were also received individually like the consumer participants. They received neither cocoa nor a placebo and were given the same confidentiality instructions. Throughout the study, no participant had to change his usual level of physical activity or diet.

### 2.5 Statistical analysis

#### 2.5.1 Measured and calculated parameters

**FIGURE 2:**
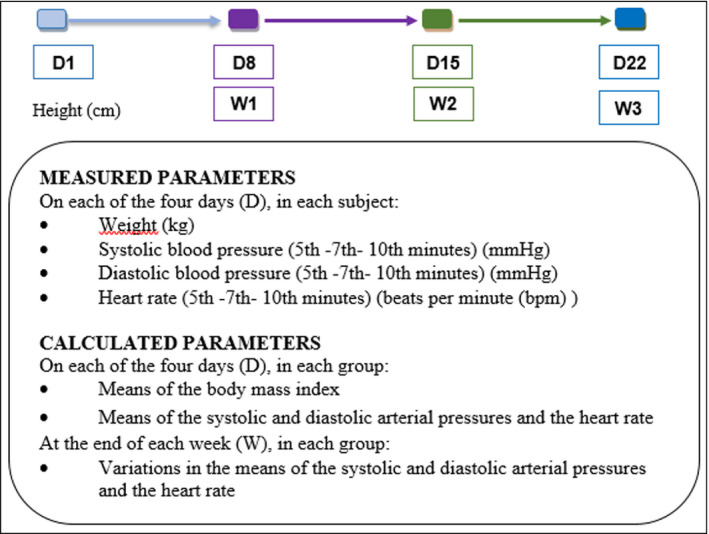
Parameters measured and calculated on each day and at the end of each week. D1: basic measurement without cocoa intake on the first day; D8: eighth day; D15: fifteenth day; D22: twenty‐second day; W1: end of the 1^st^ week; W2: end of the 2nd week; W3: end of the 3rd week

For each participant at each of the four visits, the individual averages of SBP, DBP, and HR were obtained from the three measurements taken at the 5th, 7th, and 10th minutes of rest (Figure 2). In each group, the mean SBP, DBP, and HR were established from the individual averages (Figure 2). The mean BMI of the groups were calculated from the individual values of height measured on D1 and weight measured on the day of assessment. The changes in SBP, DBP, and HR from day 8 to day 1, day 15 to day 1 and from day 22 to day 1 were calculated by the difference between the means of the parameters (SBP, DBP and HR) to these days, in each group (Figure 2). The comparisons of SBP, DBP and HR means at D1, D8, D15, and D22, and the comparisons of the changes of these three parameters at the end of W1, W2, and W3 were made within groups and between groups.

#### 2.5.2 Statistical analyses

The data obtained were imported, categorized, and analyzed with R version 4.0.2. Since the distribution of groups does not always follow a normal distribution, comparative analyses of the means were made with Kruskall‐Wallis test for comparisons of means between groups, followed by Dunn test. Friedman rank test was applied to compare the weekly evolution of parameters in the groups over the three weeks, followed by Friedman Conover test. These tests that evaluated the changes in parametric values over time were bi‐sided. For two‐to‐two comparison, we used Wilcox test (one‐sided test). Multiple linear regression was used to estimate the effect of cocoa dose on changes in SBP, DBP, and HR at the end of each week in the 10, 5, and 2 g groups compared to controls. The analyses were carried out with type I error of 0.05.

### 2.6 Sample size calculation

The sample size determination was based on a Student's t‐test formula for paired populations, to detect a change of at least 1 mmHg in arterial blood pressure in all cocoa groups after 1/2/3 weeks at each weekly time point with a standard deviation of 1.5 mmHg, taking into account a previous study in a similar population who consumed 5 g of Tafissa cocoa powder for three weeks (Balayssac‐Siransy et al., 2018). The minimum required sample size calculated was 21 subjects per group.

## 3 RESULTS

### 3.1 Body mass index

The BMI of controls and consumers was not statistically different (Table 2).

**TABLE 2:** Body mass index of control and consumer participants

Groups	Means of body mass index *(kg*/*m^2^)*				p
	D1	D8	D15	D22	
Controls n = 24	21.5 ± 2	21.5 ± 1.9	21.4 ± 2	21.4 ± 2	0.66
C 2 g n = 32	21.3 ± 2	21.4 ± 1.9	21.5 ± 1.9	21.5 ± 2	0.06
C 5 g n = 26	21.9 ± 1.9	22 ± 1.9	22 ± 1.8	22 ± 1.8	0.98
C 10 g n = 25	21.7 ± 2.1	21.7 ± 2	21.7 ± 2.1	21.7 ± 2.1	0.83
p	0.49	0.52	0.49	0.48	

Note

Data expressed as mean ±standard deviation.

Abbreviations: C 2 g, consumers of 2 g of cocoa; C 5 g, consumers of 5 g of cocoa; C 10 g, consumers of 10 g of cocoa; D1, basic measurement without cocoa intake on the first day; D8, eighth day; D15, fifteenth day; D22, twenty‐second day; n, number of participants; p, value of p (significant if *p* <0.05).

### 3.2 Systolic blood pressure

**FIGURE 3:**
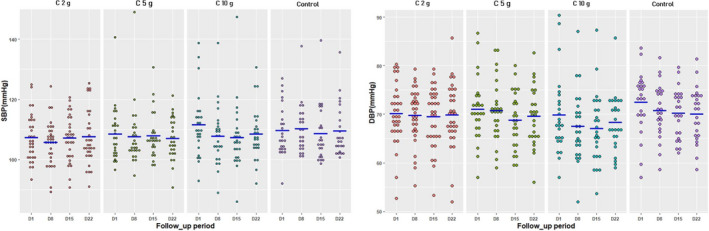
Individual values of systolic and diastolic blood pressures on each day in the four groups. SBP: systolic blood pressure; DBP: diastolic blood pressure; D1: basic measurement without cocoa intake on the first day; D8: eighth day; D15: fifteenth day; D22: twenty‐second day; C 2 g: consumers of 2 g of cocoa; C 5 g: consumers of 5 g of cocoa; C 10 g: consumers of 10 g of cocoa; blue lines expressed the averages for each day

**FIGURE 4:**
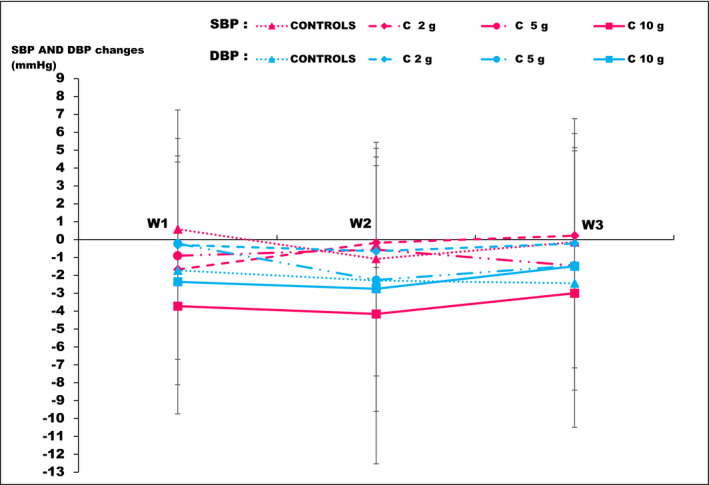
Average values of systolic and diastolic blood changes at each week in the four groups. SBP: systolic blood pressure; DBP: diastolic blood pressure; W1: end of the 1st week; W2: end of the 2nd week; W3: end of the 3rd week; C 2 g: consumers of 2 g of cocoa; C 5 g: consumers of 5 g of cocoa; C 10 g: consumers of 10 g of cocoa

The individual daily SBP values in each group have been shown in Figure 3. During the follow‐up period, there was no significant difference in means or changes in SBP among the four groups of subjects compared (Table 3). The SBP changes of the controls and the 2 and 5 g consumers were close to each other during the three‐week follow‐up (Figure 4). They differed from those of 10 g consumers, who evolved toward more negative changes in SBP (Figure 4). The two‐by‐two comparison of SBP changes of the groups, at each of the three weeks of follow‐up, revealed significant difference in the changes in SBP between the 10 g consumers and controls in the 1st week of follow‐up (−3.72 ± 6.02 mmHg versus 0.58 ± 6.66 mmHg, *p* = 0.02) and also between consumers of 10 g and consumers of 2 g in the 2nd and 3rd weeks of follow‐up (W2: −4.16 ± 8.37 mmHg versus −0.18 ± 5.62 mmHg, *p* = 0.04; W3: −3 ± 7.49 mmHg versus 0.22 ± 6.54 mmHg, *p* = 0.04) (Table 3). Multiple linear regression showed that an increase in SBP of 1 mmHg on D1 resulted in a greater decrease in SBP in consumers of 10 g cocoa than in other consumers, at each of the three weeks (all compared to controls) (Table 4). This decrease in SBP was significant during the 1st week of follow‐up in consumers of 10 g of cocoa (−4.18 mmHg; *p* = 0.04) and in consumers of 2 g of cocoa (−3.01 mmHg; *p* = 0.01) (Table 4).

**TABLE 3:** Means and changes in means of systolic blood pressure of control and consumer participants

Groups	Systolic blood pressure *(mmHg)*								
	Means				p	Changes of the Means			p
	D1	D8	D15	D22		W1	W2	W3	
Controls n = 24	110 ± 8	110 ± 8	109 ± 9	110 ± 9	0.22	0.58 ± 6.66	−1.08 ± 6.18	−0.14 ± 6.07	0.13
C 2 g n = 32	107 ± 8	106 ± 7	107 ± 7	108 ± 9	0.42	−1.67 ± 6.01	−0.18 ± 5.62	0.22 ± 6.54	0.30
C 5 g n = 26	108 ± 9	108 ± 10	108 ± 7	107 ± 7	0.54	−0.90 ± 6.86	−0.56 ± 6.36	−1.466.98	0.88
C 10 g n = 25	112 ± 11	108 ± 10	107 ± 11	109 ± 9	0.05	−3.72 ± 6.02*	−4.16 ± 8.37**	−3 ± 7.49**	0.47
p	0.37	0.24	0.95	0.78		0.22	0.28	0.31	

Note

Data expressed as mean ± standard deviation.

Abbreviations: C 2 g, consumers of 2 g of cocoa; C 5 g, consumers of 5 g of cocoa; C 10 g, consumers of 10 g of cocoa; D1, basic measurement without cocoa intake on the first day; D8, eighth day; D15, fifteenth day; D22, twenty second day; W1, end of the 1st week; W2, end of the 2nd week; W3, end of the 3rd week; n, number of participants; p, value of p (significant if *p* < 0.05).

* Significant difference between C10 g and controls (at W1 *p* = 0.02)

** Significant difference between C 10 g and C 2g (at W2 *p =* 0.04; at W3 *p* = 0.04).

**TABLE 4:** Regressions of variations in systolic and diastolic blood pressures in consumers in relation to controls at the end of each week

Weeks	Groups	Systolic blood pressure		Diastolic blood pressure	
		Coefficient of variations in mmHg (in relation to controls)	p	Coefficient of variations in mmHg (in relation to controls)	p
W1	C 2 g	−3.01	0.04	0.42	0.73
	C 5 g	−2.35	0.13	0.86	0.51
	C 10 g	−4.17	0.01	−1.74	0.19
W2	C 2 g	0.15	0.92	0.49	0.71
	C 5 g	0.03	0.98	−0.68	0.63
	C 10 g	−2.70	0.12	−1.71	0.23
W3	C 2 g	−0.45	0.78	1.08	0.41
	C 5 g	−1.73	0.31	0.27	0.84
	C 10 g	−2.20	0.20	−0.31	0.82

Note

Data expressed as p‐value.

Abbreviations: W1, end of the 1st week; W2, end of the 2nd week; W3, end of the 3rd week; C 2 g, consumers of 2 g of cocoa; C 5 g, consumers of 5 g of cocoa; C 10 g, consumers of 10 g of cocoa; bpm, beats per minutes; p, value of p (significant if *p *< 0.05).

### 3.3 Diastolic blood pressure

In each group, the individual daily DBP values were presented in Figure 3. During the study period, there was no significant difference in the means and changes of DBP among the four groups (Table 5). The curves of the changes in DBP for the three groups of consumers presented the same pattern (Figure 4). Multiple linear regression showed that an increase in DBP from 1 mmHg on D1 resulted in a greater decrease in DBP in consumers of 10 g of cocoa than in other consumers, all compared to controls, without statistical significance (Table 4).

**TABLE 5:** Means and changes in means of diastolic blood pressure of control and consumer participants

Groups	Diastolic blood pressure (mmHg)								
	Means				p	Changes of the Means			p
	D1	D8	D15	D22		W1	W2	W3	
Controls n = 24	73 ± 6	71 ± 6	70 ± 5	70 ± 6	0.20	−1.72 ± 4.97	−2.29 ± 5.33	−2.44 ± 5.97	0.97
C 2 g n = 32	70 ± 6	70 ± 6	69 ± 6	70 ± 7	0.92	−0.30 ± 4.98	−0.65 ± 5.27	−0.22 ± 5.36	0.78
C 5 g n = 26	71 ± 6	71 ± 6	69 ± 6	70 ± 6	0.03	−0.23 ± 5.89	−2.26 ± 6.39	−1.46 ± 6.42	0.02
C 10 g n = 25	70 ± 8	68 ± 7	67 ± 7	68 ± 6	0.46	−2.36 ± 5.75	−2.75 ± 6.85	−1.49 ± 5.68	0.42
p	0.14	0.11	0.20	0.57		0.59	0.64	0.53	

Note

Data expressed as mean ± standard deviation.

Abbreviations: C 2 g, consumer of 2 g of cocoa; C 5 g, consumer of 5 g of cocoa; C 10 g, consumer of 10 g of cocoa; D1, basic measurement without cocoa intake on the first day; D8, eighth day; D15, fifteenth day; D22, twenty‐second day; W1, end of the 1^st^ week; W2, end of the 2^nd^ week; W3, end of the 3^rd^ week; n, number of participants; p, value of p (significant if *p* < 0.05).

### 3.4 Heart rate

In the 3rd week of follow‐up, intergroup comparison showed a significant difference in the changes in HR between consumers of 2, 10, and 5 g of cocoa when each of them was compared to the controls (*p* = 0.001; *p* = 0.003; *p* = 0.001, respectively) (Table 6). During the three weeks of follow‐up, the HR of the 10 and 5 g consumers and of the controls showed negative and overlapping changes in HR. In consumers of 2 g, increasing changes in HR were observed to be positive from the 2nd week of follow‐up. The two‐by‐two comparison of the changes in the HR of the groups revealed a significant difference in these changes between consumers of 2 g and consumers of 5 g (4.26 ± 7.72 bpm versus −2.35 ± 9.18 bpm) and 10 g (4.26 ± 7.72 bpm versus −2.68 ± 8.01 bpm) at the 3rd week of follow‐up (Table 6).

**TABLE 6:** Means and changes in means of heart rate among control and consumer participants

Groups	Heart rate *(bpm)*								
	Means				p	Changes of the Means			p
	D1	D8	D15	D22		W1	W2	W3	
Controls n = 24	70 ± 7	67 ± 9	67 ± 9	68 ± 12	0.51	−3.17 ± 7.67	−2.58 ± 8.35	−2.29 ± 11.94	0.96
C 2 g n = 32	65 ± 9	63 ± 9	64 ± 8	69 ± 8	<0.001	−1.79 ± 5.67	0.55 ± 5.01	4.26 ± 7.72	< 0.001
C 5 g n = 26	67 ± 9	65 ± 9	64 ± 8	65 ± 7	0.79	−1.81 ± 7.45	−2.87 ± 7.78*	−2.35 ± 9.18*	0.89
C 10 g n = 25	66 ± 9	64 ± 11	64 ± 9	63 ± 8	0.23	−1.73 ± 6.13	−1.99 ± 5.82	−2.68 ± 8.01**	0.70
P	0.07	0.31	0.54	0.05		0.87	0.16	0.002	

Note

Data expressed as mean ± standard deviation.

Abbreviations: C 2 g, consumers of 2 g of cocoa; C 5 g, consumers of 5 g of cocoa; C 10 g, consumers of 10 g of cocoa; D1, basic measurement without cocoa intake on the first day; D8, eighth day; D15, fifteenth day; D22, twenty‐second day; W1, end of the 1st week; W2, end of the 2nd week; W3, end of the 3rd week; n, number of participants; p, value of p (significant if *p* < 0.05).

* significant difference between C 5 g and C 2 g (at W2 *p *= 0.04; at W3 *p *= 0.001)

** significant difference between C 10 g and C 2 g (at W3 *p *= 0.002).

## 4 DISCUSSION

The general objective of this study was to evaluate, over three weeks, the dose‐effect relationship between daily consumption of different doses (2, 5, and 10 g) of the same 100% cocoa powder and changes of SBP and DBP in young, healthy, black African males. This study, which compared four groups of healthy young subjects, including three groups of consumers of the same 100% cocoa‐based product at different doses, seems to be the first in healthy black African populations.

This work made it possible to identify a non‐linear dose‐effect relationship between cocoa consumption and changes in SBP. In fact, a significantly greater decrease in SBP was found in subjects who consumed 10 g of cocoa, compared to consumers of the lowest amount of cocoa (2 g) and controls. No significant difference was observed between consumers of 10 and 5 g of cocoa. During follow‐up, decrease in DBP was observed in the four groups of subjects, without significant difference.

### 4.1 Systolic blood pressure

In the present study, consumers who received the highest amount of cocoa (10 g per day) had the greatest reductions in SBP. These decreases were noted in relation to each of the other three groups of subjects, at each of the three weeks of follow‐up. They were significant when compared to those of consumers of the smallest amount of cocoa (2 g) and controls. Consumers of 2 and 5 g of cocoa and controls exhibited statistically similar changes in SBP at the end of the three‐week follow‐up. The daily servings of 10, 5, and 2 g of cocoa contained 1,680, 840, and 336 mg of flavonoids, respectively.

Several authors have researched the effects of cocoa polyphenols by comparing only two groups of normotensive subjects who were differentiated by the amount of polyphenols or flavanols received and sometimes by the type of product received (Al‐Faris, 2008; Almoosawi et al., 2012; Balayssac‐Siransy et al., 2018; Davison et al., 2008; Engler et al., 2004; Fraga & Oteiza, 2011; Grassi et al., 2005; Massee et al., 2015; Murphy et al., 2003; Nickols‐Richardson et al., 2014; Njike, 2011; Ried et al., 2009; Sansone et al., 2015; Shiina et al., 2009; Siransy‐Balayssac et al., 2021). The amounts of flavanols administered to consumers who received the highest doses of cocoa varied from study to study, ranging from 168 to 902 mg of flavanols per day. The other groups, called controls, received smaller daily doses of cocoa, which contained 0–36 mg of flavanols. These works mentioned above were carried out among Caucasian or black African subjects aged less than 50 years. The results of these works showed a negative or positive difference (corresponding respectively to a decrease or an increase) between SBP changes of the consumers of the largest amount of cocoa and those of the control subjects, between −7.1 and 6.29 mmHg. According to the authors, it was therefore a decrease (Al‐Faris, 2008; Balayssac‐Siransy et al., 2018; Davison et al., 2008; Fraga & Oteiza, 2011; Grassi et al., 2005; Murphy et al., 2003; Njike et al., 2011; Sansone et al., 2015; Siransy‐Balayssac et al., 2021) or an increase (Engler et al., 2004; Massee et al., 2015; Nickols‐Richardson, 2014; Njike, 2011; Ried et al., 2009; Shiina et al., 2009) of SBP in large‐quantity consumers compared to small‐quantity consumers or controls. Only few authors have compared three or four groups of consumers. Mastroiacovo et al. (Mastroiacovo et al., 2015) conducted a double‐blind, controlled, parallel‐arm study in 90 elderly subjects (men and women, some with hypertension). They were randomly assigned into three groups to consume a dairy‐based cocoa drink containing 993 mg (high), 520 mg (intermediate), or 48 mg (low) cocoa flavanols, once daily for eight weeks. At the end of follow‐up, the researchers noted significant SBP reduction among high‐ and intermediate‐quantity subjects compared to low‐quantity subjects (−7.83 ± 0.56 and −6.8 ± 0.59 versus −1.6 ± 1.06; *p *< 0.05). Davison et al. (Davison et al., 2010) matched 52 male and post‐menopausal female adults with high normal blood pressure or untreated mild hypertension into four treatment groups. They were randomly assigned to daily consume reconstituted cocoa beverages containing 33, 372, 712, or 1,052 mg of flavanols, for six weeks. No evidence of dose‐related response was seen. There were no dose x time interactions and no significant difference between groups for seated SBP (*p* = 0.86). Nested analysis (time nested in dose) revealed significant dose effects for 24 hour, daytime, and night time SBP (*p* = 0.001; *p* = 0.02; *p* = 0.003 respectively). Post hoc analysis showed that for the 1,052 mg flavanol dose, the reduction in 24 h SBP (*p *< 0.02) was significantly different from that of all other doses.

The SBP decrease in higher‐quantity consumers compared to control subjects supports the importance of the quantity of flavanols consumed. Indeed, the decrease in SBP would be greater, the higher the quantity of polyphenols (flavonoids) consumed (Al‐Faris, 2008). The flavanols in cocoa stimulate the enzyme endothelial NO synthase, thereby promoting increased endothelial production of NO. NO causes vasodilation with a consequent drop in blood pressure. In addition, cocoa flavanols inhibit, on the one hand, angiotensin I converting enzyme (therefore the formation of angiotensin II, a powerful vasoconstrictor), and on the other hand, the vasoconstrictor action of endothelin 1, thus causing a drop in blood pressure (Lamuela‐Raventos et al., 2005; Ried et al., 2017). The changes in significance from one study to another could be due to the amount of sugar in the product consumed, intergroup difference in the amount of flavanols received, or type of blood pressure measurement (seated position at rest or 24‐h outpatient measurement). Indeed, the addition of sugar to cocoa would cancel the vasodilator effects of flavanols (Ried et al., 2017). For Fraga et al. (Fraga & Oteiza, 2011), the amounts of sugar in milk chocolate were respectively equal to 54.2 and 60.2% per group. Matroiacovo et al. (Mastroiacovo et al., 2015) as well as Davison et al. (Davison et al., 2010) used products that contained around 17 g of sugar per 58 g serving of drinks (around 30%). Furthermore, the smaller the difference between flavanol quantities administered to consumers and to controls, the closer the changes in SBP of these groups would be (Ried et al., 2017). In our study, this difference was between 1,680 and 840 mg against 168 mg in Fraga et al.'s study (Fraga & Oteiza, 2011), 945–472 mg in Mastroiacovo et al.'s study (Mastroiacovo et al., 2015), and 1,019 mg in Davison et al.'s study (Davison et al., 2010).

### 4.2 Diastolic blood pressure

At the 3‐week follow‐up, the means and changes in DBP were not statistically different among the four groups of consumers and controls.

In many works (Al‐Faris, 2008; Almoosawi et al., 2012; Balayssac‐Siransy et al., 2018; Davison et al., 2008; Engler et al., 2004; Fraga & Oteiza, 2011; Grassi et al., 2005; Heiss et al., 2015; Massee et al., 2015; Murphy et al., 2003; Neufingerl et al., 2013; Nickols‐Richardson et al., 2014; Njike et al., 2011; Ried et al., 2009; Sansone et al., 2015; Shiina et al., 2009; Siransy‐Balayssac et al., 2021), the effects of cocoa polyphenols on DBP were investigated by comparing two groups of subjects. The daily amount of flavanols administered to consumers of the highest amounts of cocoa was between 168 and 902 mg. The control groups received between 0 and 36 mg of flavanols per day. The results made it possible to note a difference in the DBP between the consumers of the largest amounts of cocoa and the control subjects, between −7.6 and 1.5 mmHg. While some of the authors found a decrease in DBP (Al‐Faris, 2008; Almoosawi et al., 2012; Davison et al., 2008; Fraga & Oteiza, 2011; Grassi et al., 2005; Heiss et al., 2015; Massee et al., 2015; Murphy et al., 2003; Neufingerl et al., 2013; Njike et al., 2011; Sansone et al., 2015; Siransy‐Balayssac et al., 2021), others found an increase (Balayssac‐Siransy et al., 2018; Engler et al., 2004; Nickols‐Richardson et al., 2014; Ried et al., 2009; Shiina et al., 2009) among large‐quantity cocoa consumers compared to small‐quantity cocoa consumers.

Few works have compared changes in DBP among three groups of consumers receiving different daily doses of flavanols. Mastroiacovo et al. (Mastroiacovo et al., 2015), at the end of eight weeks of following up three groups who were receiving three doses of flavanols, found no significant difference between DBP decrease among the three groups (−4.77 ± 0.37 and −3.2 ± 0.36 versus −1.57 ± 0.61; *p *> 0.05). Davison et al. (Davison et al., 2010) randomized 52 subjects into four treatment groups (33, 372, 712 or 1,052 mg per day of flavanols, for 6 weeks). No evidence of dose‐response was seen. There were no dose x time interactions and no significant differences between groups for seated DBP (*p* = 0.45). Nested analysis (time nested in dose) revealed significant dose effects for 24‐h DBP (*p* = 0.002). Post hoc analysis showed that for the 1,052 mg flavanol dose, the reductions in 24‐h DBP (*p* < 0.04) were significantly different from all other doses.

Several factors may be responsible for variable intergroup differences, ranging from decrease to increase in DBP, to similarity, among high‐quantity cocoa consumers compared to smaller‐quantity cocoa consumers. DBP is a hemodynamic parameter that is dependent on peripheral arteriolar resistance and on the condition of the wall of the large arterial trunks (Asmar, 2007).

### 4.3 Heart rate

In our work, in consumers of 10 and 5 g of cocoa as well as in controls, a statistically similar drop in HR was found at the end of each of the three weeks. Consumers of 2 g of cocoa powder experienced an increase in HR.

In most of the previous studies (Al‐Faris, 2008; Balayssac‐Siransy et al., 2018; Davison et al., 2008; Fraga & Oteiza, 2011; Heiss et al., 2015; Massee et al., 2015; Murphy et al., 2003; Neufingerl et al., 2013; Siransy‐Balayssac et al., 2021), the effects of cocoa polyphenols on HR were investigated by comparing two groups of subjects who consumed different quantities of polyphenols or flavanols: 168‒902 mg of flavanols per day in heavy consumers and from 0 to 36 mg of flavanols per day in control subjects. The populations of these studies were composed of Caucasian or black African subjects, of both genders or only males, under 50 years of age. The results showed difference in HR between the consumers of the largest quantity of cocoa and the control subjects included, between −7 and 1 beat per minute, ranging from a decrease (Davison et al., 2008; Murphy et al., 2003) to an increase (Al‐Faris, 2008; Balayssac‐Siransy et al., 2018; Fraga & Oteiza, 2011; Heiss et al., 2015; Massee et al., 2015; Neufingerl et al., 2013; Siransy‐Balayssac et al., 2021) in HR of consumers of large quantities of cocoa in relation to controls. However, Davison and al (Davison et al., 2010), in a study of four groups that consumed different doses of cocoa, did not find significant effect on HR.

As cocoa is a fruit recognized for its richness in flavanols and minerals, including potassium, it is not clear whether the effects of cocoa on HR is due to flavanols or to potassium in cocoa. A study that examines the relationship between potassium in cocoa and HR would help to better understand changes in HR that occur with consumption of cocoa. In addition, larger population and assessment of individual variability of HR in black African population would help.

### 4.4 Methodological limitations

Only males were included in this study because hormonal fluctuations in women during the menstrual cycle may influence arterial blood pressure (Balayssac‐Siransy et al., 2014; Moran et al., 2000). Therefore, to investigate the effects of cocoa on the cardiovascular system of females, a different study protocol will be needed, and it should include the menstrual phases of two successive cycles.

The choice of participants aged 18–30 years was motivated by the data in the literature, according to which the drop in SBP and DBP induced by cocoa consumption were greater in subjects under 50 years of age because of the arterial stiffness that occurs with age (Ried et al., 2017; Taubert et al., 2007).

The control group did not ingest a placebo solution. The single‐blind study design could constitute a bias because it tends to induce greater reduction in arterial blood pressures compared to double‐blind studies, although the difference is not statistically significant according to the data in the literature (Ried et al., 2017).

Selection criteria and the weekly food sheet filled in by the subjects were used to detect confounding factors, including drugs that have an effect on blood pressure, dietary changes, major stress, or concomitant pathology.

In this work, endothelial function was not assessed. Indeed, NO measurement would have made it possible to establish correlations between the amount of flavanols consumed, changes in blood level of NO, and changes in blood pressure.

## 5 CONCLUSION

This experimental study, which was conducted in a healthy young black African male population, investigated the dose‐effect relationship between regular consumption of different doses of the same 100% cocoa powder and changes in systolic and diastolic arterial pressures. Carried out over three weeks, the study found significant reduction of SBP in the consumers of the largest quantity of cocoa (10 g) with no linear relationship between cocoa doses and SBP changes. Changes in DBP were statistically similar for the four groups of subjects. The likely effect of individual differences on blood pressure changes suggests the potential benefit of a cross‐over study in which the consumer subject is his own control.

## CONFLICT OF INTEREST

The authors declare that the research was conducted in the absence of any commercial or financial relationship that could be construed as a potential conflict of interest.
